# Providing technical assistance: lessons learned from the first three years of the WHO Adolescent and Youth Sexual and Reproductive Health and Rights Technical Assistance Coordination Mechanism

**DOI:** 10.1186/s12978-024-01800-6

**Published:** 2024-06-19

**Authors:** Bruce Dick, Marina Plesons, Callie Simon, Jane Ferguson, Ahmed Kassem Ali, Venkatraman Chandra-Mouli

**Affiliations:** 1 WHO TA Mechanism Secretariat, WHO, Geneva, Switzerland; 2 Consultant Adolescent Health, Geneva, Switzerland; 3 University of Miami, Miller School of Medicine, Miami, USA; 4 Senior Adolescent Sexual and Reproductive Health Advisor and Team Lead, Save the Children, Washington DC, USA; 5 WHO TA Mechanism, Geneva, Switzerland; 6 Department of Sexual and Reproductive Health & Research, and TA Mechanism Secretariat, WHO Geneva, Geneva, Switzerland; 7 Adolescent Sexual and Reproductive Health, Department of Sexual and Reproductive Health & Research, and WHO TA Mechanism Secretariat, WHO, Geneva, Switzerland

## Abstract

Young people’s sexual and reproductive health (SRH) continues to be a major challenge in low and middle-income countries, with implications for public health now and in the future. Fortunately there is a growing array of evidence-based interventions, and commitments from governments, development partners and donors, to support programmes that aim to improve young people’s SRH.

However, in some situations, the technical assistance that governments feel that they need to strengthen and implement national policies and strategies, to move from words to action, is not available. The WHO Adolescent and Youth Sexual and Reproductive Health and Rights (AYSRHR) Technical Assistance (TA) Coordination Mechanism was initiated to help fill this technical assistance gap; to respond to TA requests from ministries of health in ways that are timely, efficient, effective and contribute to strengthening capacity.

This paper describes the process of developing the Technical Assistance Coordination Mechanism (TA Mechanism) and the outcomes, experiences and lessons learned after three years of working. It triangulates the findings from a preliminary review of the literature and discussions with selected key informants; the outcomes from a series of structured review meetings; and the documented processes and results of the technical assistance provided to countries.

The lessons learned focus on three aspects of the TA Mechanism. How it was conceptualized and designed: through listening to people who provide and receive AYSRHR TA and by reviewing and synthesizing past experiences of TA provision. What the TA Mechanism has achieved: a standardized process for TA provision, at different stages for a range of AYSRHR issues in ten countries in three geographic regions. And what worked well and what did not: which common challenges was the TA Mechanism able to address and which ones persisted despite efforts to avoid or resolve them. The paper ends with the implications of the lessons learned for future action.

## Introduction and context

This paper describes how an innovative mechanism, housed by the World Health Organization’s Department of Sexual and Reproductive Health and Research and aiming to provide technical assistance on Adolescent and Youth Sexual and Reproductive Health and Rights (AYSRHR), was conceived and designed; how it was operationalized and what it achieved over three years; and what lessons were learned from those aspects of the mechanism that worked well and those that did not.

In the last five years of the Millennium Development Goals era and in the eight years of the Sustainable Development Goals era, aspects of AYSRHR are on the priority health, development and human rights agendas globally, regionally and nationally in a growing number of countries. These include preventing HIV infection and HIV-related mortality and morbidity, preventing early pregnancies and childbearing and the health and social consequences associated with them, and preventing and mitigating the negative effects of harmful traditional practices such as child marriage and female genital mutilation [1].

On the positive side, there is more money to support AYSRHR programmes in low- and middle-income countries than ever before; there is a growing body of epidemiologic data and evidence from research studies and programmatic experience; and tools are available to support policy and programme design, execution and assessment. On the negative side, discomfort about addressing the sensitive matters of AYSRHR and weak capacity, especially in governments, hinders the translation of evidence to action using the commitments that have been made and the funds that are available. There is frequently only token adolescent involvement, if at all, and nongovernmental organizations with a track record in AYSRHR are left out of government initiatives. As a result, national policies and strategies are often poorly designed, weakly implemented and monitored, and lessons are not systematically documented and shared [2].

However, when government officials in Ministries of Health want to obtain technical support, they face many challenges in doing so. For example, TA is often not country driven: Ministries of Health are not always in the “driver’s seat” for decisions about the technical assistance that they need to help move their intentions from words to action; and the priorities of funding agencies and NGOs may take precedence [3].

To address this challenge, the Bill and Melinda Gates Foundation supported WHO to set up and run an AYSRHR Technical Assistance Coordination Mechanism (TA Mechanism) to support countries make full use of the growing commitment and resources for AYSRHR, by helping them move from ‘ready and wanting to act’, to implementing effective interventions. It aimed to do so by coordinating the provision of high-quality TA that is timely, effective, efficient, and contributes to capacity development and mentoring; and that responds to the expressed needs of selected countries for planning, implementing, monitoring, evaluating, reviewing, and documenting their AYSRHR programmes. This was part of the Foundation’s support to WHO to support ministries of health realize the commitments to accelerate access to and use of contraceptives in all individuals of reproductive age, within the broader framework of the Sustainable Development Goals and Universal Health Coverage.

This paper describes the processes used to develop the TA Mechanism; it examines what the Mechanism set out to achieve and what it in fact did; it reviews the experiences of three years of implementation and considers what worked well and what did not. It then discusses the implications for both the work of the Mechanism in the future and for the field more widely. It focuses on three questions:

1. How was the TA Mechanism conceptualized and designed?
2. What has the TA Mechanism achieved during its first three years?
3. What worked well and what did not, and what are the implications of the lessons learned for future action?

## Methods

The data collection and data analysis methods used in relation to the three objectives of the paper are described below.

### How was the TA Mechanism conceptualized and designed?

This was done using three complementary methods:

- A rapid scoping literature review to identify reports of experiences and lessons learned in providing technical assistance to countries. The review was based on citations in Google Scholar
- Interviews and group discussions with individuals with experience in providing and/or receiving TA, to learn their perceptions on what was needed, and what was not.
  Interviews and group discussions were undertaken opportunistically as part of country visits (to India, Liberia, Nepal) and during multi-country workshops organized by the WHO Family Planning Umbrella Project, Family Planning 2020 (now Family Planning 2030), the Global Programme to Accelerate Action to End Child Marriage, and the Global Fund to Fight AIDS, Tuberculosis and Malaria. Information was also obtained from meetings and calls with key partners (e.g. Bill and Melinda Gates Foundation, USAID, the Global Financing Facility, UNAIDS, UNICEF, and UNFPA), and from internal consultations within WHO.
- A co-creation meeting with a group of non-governmental organizations (NGOs) with expertise and experience of developing and implementing AYSRHR programmes in low- and middle-income countries (LMICs), who were invited to WHO Geneva on 15-17 April 2019, along with UNFPA and FP2030 [4], to build a shared understanding of principles and approaches for the TA Mechanism.

### What did the TA Mechanism achieve during its first three years?

Information was gathered from the following three sources: minutes of meetings, reports submitted by partner organizations which provided technical support, and reports prepared by the TA Mechanism Secretariat.

### What worked well and what did not, and what are the implications of the lessons learned for future action?

Information on this was gathered using the following methods:

- An internal review meeting in November 2019, involving the TA Mechanism Secretariat and staff from the overall WHO FP Accelerator project, to discuss the focus of the on-going work, progress and initial challenges.
- Notes for the record of routine meetings with Partner Organizations, WHO colleagues and key collaborators including BMGF and USAID; and *ad hoc* meetings with Partner Organizations and ministries of health to review progress and respond to issues requiring discussion and solutions.
- A virtual rapid end-of-the-year reflection with Partner Organizations in December 2020 to assess progress and identify issues of concern that required further action, and an internal in-depth brainstorming review of the TA Mechanism by the Secretariat in March 2021 for a frank, in-depth discussion at the end of the first year on a range of issues that it would not have been possible to discuss in a more open setting.
- A structured TA Mechanism review meeting involving Partner Organizations and beneficiaries, notably ministries of health, in June 2021 [5]. This virtual review meeting took place over two days and involved countries that had requested TA, Partner Organizations who had been involved with responding to TA requests, other partners such as FP2030 and BMGF, the TA Mechanism Secretariat and other members of the WHO FP Accelerator Project.
- End-of-year comments during the December 2021 monthly standing call, when the opportunity was taken to obtain some rapid reflections from the Partner Organizations on their involvement with the TA Mechanism and activities during the past twelve months.
- Deliverables produced as a result of the TA that had been provided.

## Findings

### How was the TA Mechanism developed?

The review that we began our work with identified only a handful of assessments of TA directed to improving the SRHR of adolescents and young people, although it also identified a number of assessments/evaluations of TA provided on other topics in response to the needs of other population groups. The meetings with the key informants provided an overview of existing mechanisms that governments (including, but not limited to ministries of health), and technical and funding support agencies use to request and provide TA, and an opportunity to synthesize some lessons learned. Put together, these helped identify a number of factors that needed to be taken into consideration during the development of the TA Mechanism and the drafting of the Standard Operating Procedures (SOP).

First, that there are a range of reasons for TA to be requested, or offered, for example to fill staffing gaps, to provide technical inputs on various issues and to strengthen capacity. It may be required for short-term specific programme needs or over the longer-term life of a project/programme.

Secondly, there are different formats and processes through which TA can be provided, all of which have advantages and disadvantages depending on the expectations of the TA and the resources available to carry it out. These include setting up communities of practice to share experiences, expertise, and programme support tools; organizing webinars that provide technical updates and opportunities for questions and answers; facilitating visits by one or more selected staff to other countries where the technical or programmatic issues of concern have been effectively responded to; organizing for individuals from the requesting country to take part in relevant training programmes; or having individuals or teams from within the country requesting the TA, or from outside, provide support, either on a “fly-in fly-out” basis or in ways that provide longer-term support.

Thirdly, there are a number of factors related to the individuals and/or organizations that are responsible for the TA that need to be considered. For example, TA providers are likely to be influenced by their past approaches to providing TA, and by the mandates, structures, priorities and governance of the organizations that they work for (i.e. what they can and cannot do, what they are interested in, how they are able to provide the TA). In addition, the inputs from the global, regional and national levels of organizations need to be considered, in terms of both the selection of the person who will carry out the TA and also processes for reviewing the products of the TA, which may be beneficial, but may also be a source of delays and disagreements.

The review also pointed out that there are a number of other common challenges facing people and organizations providing TA. For example, the Terms of Reference (TOR): are they clear and do they really reflect what it is that the country wants; is there a good match between what the country needs and the expertise and priorities of the organization providing the TA; are there opportunities for the TA provider to work with the country requesting the TA to refine the TOR; and is it likely that there will be a sufficient “dose” of TA to have the desired effects?

Another example of the challenges that those providing TA face is the outputs of the TA: the importance of developing consensus across different providers of TA on the evidence-base for action and the implications of this for programme priorities; and the need to have agreement about what is really useful and likely to be used in relation to any recommendations that might be made. Consideration also needs to be given to the systems that are adopted for monitoring milestones and the quality of the TA that is provided, and for making the links between different but related aspects of the TA, both technical (e.g., HIV and SRH) and programmatic (e.g., focusing on specific outcomes and dealing with the need to strengthen health systems more generally).

These findings were used to draft an SOP for the TA Mechanism setting out the guiding principles, the overall approach and the detailed working methods. This document was tabled and discussed in the co-creation meeting, and led to the development of an agreed *modus operandi* for moving ahead.

### What did the TA Mechanism achieve?

The TA Mechanism was initiated with the following aims:

- To provide TA to ministries of health that will help them achieve the goals/commitments that they have defined to improve AYSRHR (with a particular focus on contraceptive uptake);
- To provide the TA in ways that are timely, effective, efficient, innovative and contribute to capacity development;
- To contribute to overall thinking and lessons learnt about the provision of TA.

A number of principles were identified to guide the TA Mechanism, based on the preparatory activities that were carried out, which were incorporated into the SOP:

**Table:** 

What issues would the TA Mechanism address? Increasing contraception uptake should be a central component of any request, in order for the TA Mechanism to limit the types of requests that it would respond to (i.e., to manage demand and to ensure quality responses). However, the TA Mechanism would also strive to find a balance between the attention that is given to contraceptive uptake and wider AYSRHR problems, to AYSRHR and adolescent and youth health more generally, and to AYSRHR outcomes and their underlying determinants.
Who would provide TA? Responses to TA requests would be provided through experts working with the TA Mechanism's Partner Organizations, or when such support was unavailable, through national or international consultants - with the support and facilitation of the TA Mechanism Secretariat.
How would the Partner organizations be chosen? Partner organizations would be selected based on the following criteria: a strong track record of working in the field of ASRHR in LMICs; experience in providing technical support and collaborating with a variety of stakeholders, especially governments, other non-governmental organizations, and youth-led organizations; an interest to be involved with the TA Mechanism, and staff with the experience and flexibility to provide TA as required. At the same time, efforts would be made to ensure that the organizations selected covered a range of expertise and had diverse country-level representation.
How would countries be informed about the TA Mechanism? Countries would be informed through WHO’s regional offices, through UN partners, notably UNFPA, through funding agencies such as BMGF and USAID, and mechanisms such as FP2030 and the GFF.
How would countries decide what TA to request? The countries would be self-selected - there would be no pressure on the TA Mechanism to include specific countries. The development of the requests would be led by ministries of health and involve relevant in-country stakeholders (e.g., UN organizations, civil society organizations (CSOs) and young people). The requests would be submitted to the TA Mechanism by ministries of health at national or subnational levels (i.e., the requests would be fully country-led with government buy-in and leadership).
What role would the TA Mechanism Secretariat play? The T Mechanism Secretariat would play an “honest broker” role in terms of helping to define and clarify the TA requests, as needed; to provide a sounding board for the responses as these are developed; and to play a key role in terms of quality assurance.

**Fig. 1:**
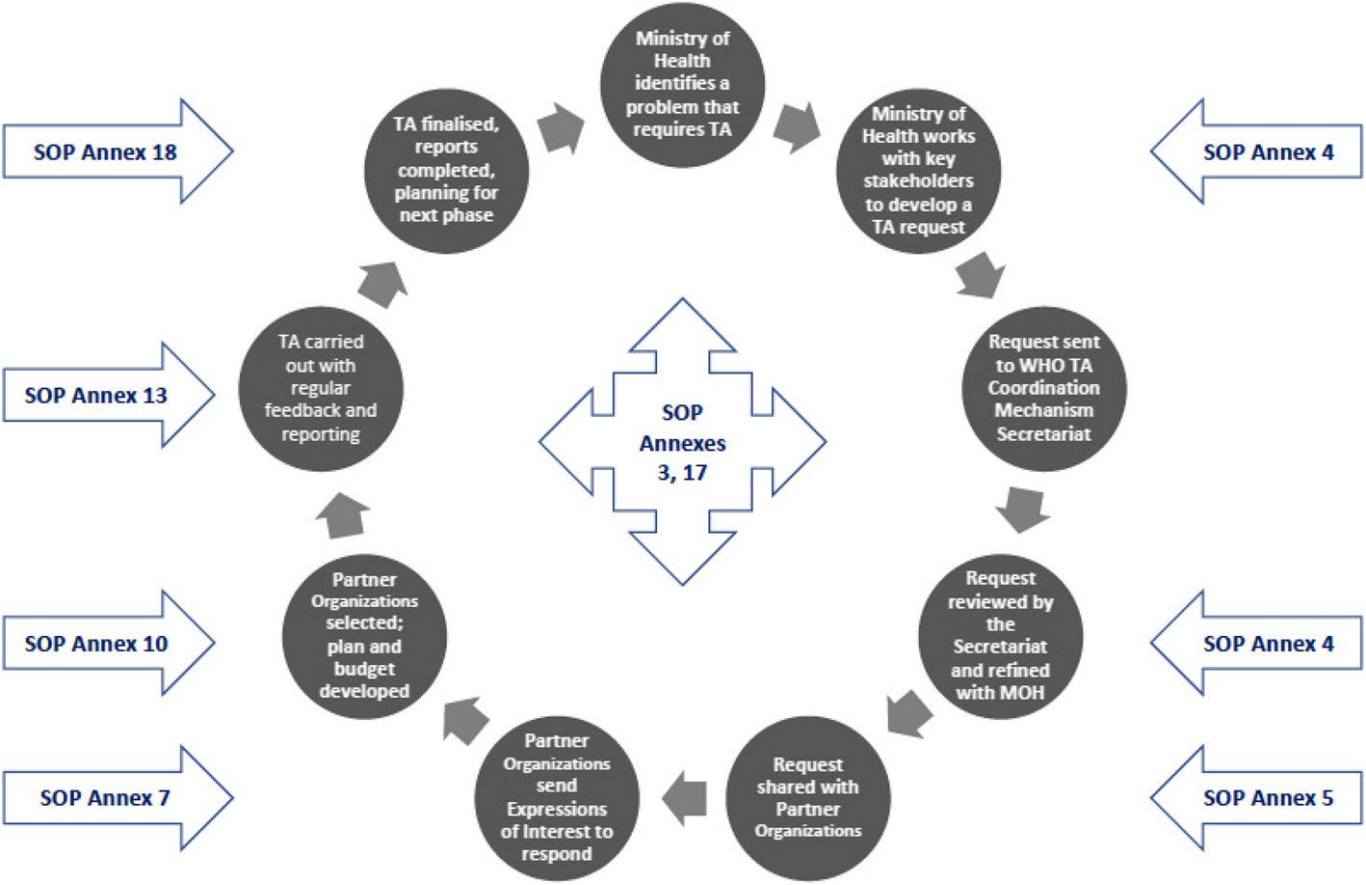
Brief overview of the life-course of a TA request and response, and links to the standard operations procedures (SOP)

The SOP [6] has guided the process of making and responding to requests for TA (see Fig. 1). This process has been added to and modified during the Mechanism’s three years of operation, based on the collective experiences gained through learning-by-doing. In addition, a number of activities were initiated by the TA Mechanism Secretariat in order to manage the process, facilitate collaboration, improve communication and maintain quality assurance. These included monthly meetings with the Partner Organizations; regular meetings with Partner Organizations and WHO Country Offices (WCOs) in countries where TA was being provided; regular meetings with the funders (BMGF), key partners such as FP2030, and WHO colleagues responsible for the overall Accelerator project; and updates for ministries of health about progress and challenges, and their inclusion in the TA plans and budgets.

As of the end of 2022 the TA Mechanism was at various stages of TA provision in 11 countries (Afghanistan, Cameroon, India, Kenya, Liberia, Malawi, Mali, Nigeria, Senegal, Sierra Leone, Uganda), with expressions of interest from an additional 4 countries (Democratic Republic of the Congo (DRC), Pakistan, Tanzania, Zambia). It had rejected requests from only two countries, as these turned out to be more requests for funding than for technical assistance (Colombia and South Africa). Table 1 provides an overview of the range of these TA requests and the responses that are currently being developed and implemented.

**Table 1:** TA Mechanism support for countries, completed and in progress (end-2022)

**Country**	**Overall objectives of the TA**	**Expected outcomes**	**Status**
Partner Organizations and consultants providing TA			
**Afghanistan**	To better understand the SRHR needs of adolescents and the status of the MOPH’s AYSRHR policies and programmes, and outline options for strengthening the national response to meeting the health needs of young people	Strengthened national policies and programmes on adolescent health, including ASRH	Phase 1 completed; planning for phase 2 is ongoing with WCO and UNFPA
Phase 1: CARE, MAMTA and a national consultant Phase 2: MSI Choices and MAMTA			
**Cameroon**	To develop a strategy and operational plan for strengthening demand generation and contraception service provision for adolescents and young adults in eight state-owned universities	Strengthened AYSRH/HIV demand generation and service provision in eight state-owned universities	Contracts and planning for Phase 1 completed, and soon to be initiated
PSI/ACMS			
**India**	To support the Himachal State Government to digitalize tools to train and retrain different cadres of health workers on adolescent health.	Strengthened capacities of health workers using distance learning techniques	Phase 1 planning meeting completed and phase 2 being initiated
MAMTA and Pathfinder International/India			
**Kenya**	To support five coastal counties to develop a regional strategy and county-level plans to address adolescent pregnancy	Strengthened county-level policies and strategies to prevent adolescent pregnancy	Phase 1 completed and findings disseminated to new Executive; planning for Phase 2 in progress
AKU, EGPAF and Pwani University			
**Liberia**	To improve health worker performance for ASRH services through the development of innovative in-service training, supervision and mentoring	Improved ASRH training programmes for health workers in terms of content and processes	Integrated plan and budget finalized and contracts completed
CARE and national consultant			
**Malawi**	To better understand why, despite significant investment and efforts on ASRHR, the country has not seen more progress in relation to adolescent contraception and early pregnancy reduction	Strengthened national policies and programmes on ASRH, with coordination by the MOHP	Phase 1 has been completed. No further action envisaged at this time.
PSI plus international and national consultants			
**Mali**	To conduct a review of the *Plan d’Action Multisectoriel Santé des Adolescents et des Jeunes* 2017-2021 and identify interventions/approaches to contribute to the development of the post-2021 plan	An assessment of the implementation of the current plan and key elements for inclusion in the next *Plan d’Action*	Contracts and planning for Phase 1 completed and about to be initiated
MSI Choices and Equipop			
**Nigeria**	Phase 1: To contribute to the development of the National Adolescent Health Policy. Phase 2: To strengthen health worker capacity; support the operationalization of relevant policies and strategies for the provision of services for adolescents at State and LGA level, including monitoring and evaluation; develop a research agenda and document/disseminate good practices in Nigeria of ADH/ASRH programming	Improved health care providers' performance; a strengthened and integrated State and LGA level SRH service provision for adolescents; an ASRH/ADH research agenda and a synthesis of lessons learned from documented best practices	Phase 1 completed. An Inception meeting for Phase 2 has been completed, and planning is ongoing
Phase 1: TA Mechanism Secretariat and an international consultant Phase 2: Pathfinder International/Nigeria, PSI, IPPF/PPFN and MAMTA			
**Senegal**	To carry out an analysis of adolescent pregnancy in the country and develop a strategy, operational plan, and M&E plan to decrease adolescent pregnancy, with a focus on married adolescents	A strategy and operational plan for meeting the needs of pregnant adolescents and first-time adolescent mothers	Phase 1 completed, awaiting plan/budget for phase 2
PATH and National Youth Association			
**Sierra Leone**	Phase 1: To develop national guidelines for health workers on providing quality care for pregnant adolescents and first-time adolescent mothers Phase 2: To develop a training module that can be incorporated into the existing relevant training programmes	Improved health workers’ capacity for providing quality of care for pregnant adolescents and first-time adolescent mothers	Phase 1: Guidelines completed Phase 2: Draft training manual finalized, awaiting field-testing and finalization
Phase 1: Save the Children Phase 2: International consultant and Save the Children			
**Uganda**	To review and harmonize current ASRH/ADH policies, strategies, guidance and standards; strengthen and integrate a focus on adolescents and youth into the national health policy and the RMNCAH strategy; identify a small number of “learning districts” to provide operational experience for the integrated adolescent and youth components of the national health policy	Harmonized ASRH/ADH policies and strategies; adolescent priorities well represented in national health policy and RMNCAH strategy; operational experience from the “learning districts”	Inception meeting about to take place; awaiting the development of an integrated TA plan to initiate Phase 1
Phase 1: PSI and PATH			

In terms of the requests, while they all include a focus on AYSRHR, there was variation in terms of a number of key variables.

The overall focus: The majority of the requests focused on analyzing the current situation with a view to developing and strengthening subsequent activities to increase contraception uptake, to improve AYSRHR and to positively impact adolescent health more generally, using AYSRHR as an entry point. Most of the requests therefore initially involved carrying out situation assessments, including desk reviews and landscape analyses, in order to provide a basis for the subsequent development of strategies and operational plans. Three of the TA requests included the development of specific products: the request from the Ministry of Health and Sanitation (MOHS) in Sierra Leone, to develop national guidance on pregnant adolescents and first-time adolescent mothers; the request from the MOH Liberia, to develop training materials and innovative approaches to training service providers to strengthen their capacity to meet the health needs of adolescents and youth, including ASRH; and the request from India, for TA to support the development of a digital e-learning course for adolescent health service providers in the state of Himachal Pradesh, based on the nationally endorsed training materials for *Rashtriya Kishor Swasthya Karyakram* (RKSK), the national adolescent health programme.

The target group: Most of the requests for TA have targeted the general population of adolescents and youth. However, the requests from Sierra Leone and Senegal specifically focused on pregnant adolescents and first-time/married adolescent mothers, and the request from Cameroon focused on young people in tertiary education settings.

In terms of the responses to these requests, again there were a number of common elements, in line with the principles outlined in the SOP.

A partnered response: The majority of the requests have involved TA from more than one Partner Organization, something that was proposed by the participants of the initial TA Mechanism co-creation planning meeting. While this has required additional time and effort to plan and coordinate the responses, and is likely to have increased the costs of the TA provided, it has proven to be a positive element of the TA Mechanism, strengthening both the quality of the responses and the collaboration between the Partner Organizations who form the core of the TA Mechanism.

In-country presence: The TA Mechanism always aimed to avoid fly-in fly-out responses to providing TA, and to maximize the contextual relevance, minimize the costs and improve the time efficiency of responses by engaging, when possible, with local partners. This was greatly assisted by the COVID-19 pandemic, which significantly limited travel over the Mechanism’s first two years of operation. It has therefore been essential to have had at least one Partner Organization that has a presence in the country requesting the TA - something that has been important for a range of reasons, from understanding the context to using existing networks to facilitate communication.

A phased approach: In general, the TA that has been provided has been planned in phases. This has been partly related to practical considerations, such as the need to keep the budgets within the limits set by WHO for individual contracts. However, there have also been technical reasons for this phased approach: it has been useful for ensuring that there is a logical progression in what is done, to provide an opportunity to review the appropriateness of the subsequent phases included in the initial plan and to make it possible for other Partner Organizations to be involved in subsequent phases if the skills that they have are more appropriate to the tasks at hand. It has also made it possible to have short-term achievements within the longer-term on-going TA. There are currently four countries initiating or undertaking phase 1 activities (Cameroon, Mali, Liberia and Uganda) and 6 countries planning or providing phase 2 TA (Afghanistan, India, Kenya, Nigeria, Senegal and Sierra Leone). With the exception of Malawi, all countries that completed phase 1 have subsequently moved to phase 2.

An impact model: An impact model was developed that could be adapted for each individual TA request, in order to focus the activities of the TA Mechanism and clarify the expectations for TA responses. By outlining what the TA Mechanism would and would not aim to achieve, and what it could and could not be responsible for doing, the impact model helped to clarify accountability and attribution. In doing so, it also sought to be explicit about those aspects of programme development and implementation for which ministries of health and other partners would be primarily responsible. For these components the TA Mechanism would only be responsible for advocacy and monitoring in relation to the overall intended impact of the technical assistance provided. Table 2 provides an example of the use of the Impact Model for Sierra Leone.

**Table 2:** The impact model – example from Sierra Leone

**Country**	**The TA Mechanism is responsible for supporting:**	**The TA Mechanism is responsible for advocating for:**	**The TA Mechanism will track to see if:**	**Intended impact of the TA Mechanism support:**
**Sierra Leone**	The development of guidelines and a related training module and desk-reference tool for integration with existing health worker training programmes, so that meeting the needs of first-time adolescent mothers becomes “everybody’s business”	Integration of the module into existing training programmes Enactment of complementary procedures that are needed to ensure health system readiness Sending a directive to sub-national authorities and through them to health facility managers	Health workers are required and trained to provide this care as per the guideline The health system makes changes to enable health workers to provide this care	Improved quality of care for pregnant adolescents and first-time adolescent mothers within the existing health care system

**Fig. 2:**
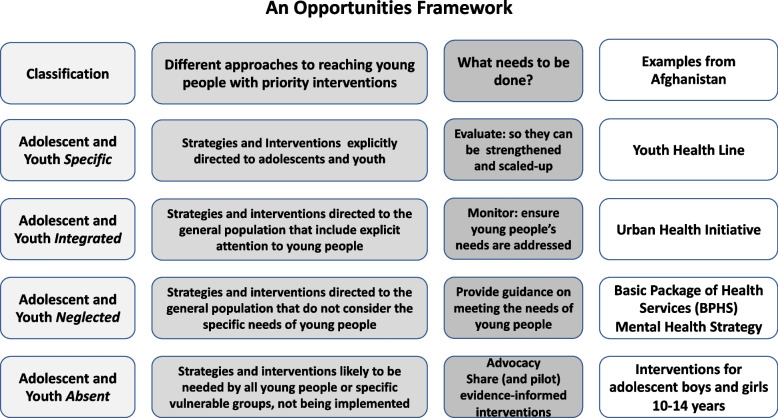
An opportunities framework for TA recommendations – example from Afghanistan

An Opportunities Framework: During the course of the three years, a number of tools were developed by the TA Mechanism Secretariat and the Partner Organizations to support the provision of TA. One of these, developed during Phase 1 of the TA response in Afghanistan, was a framework that aimed to synthesize recommendations more strategically - to move beyond the common problem of long lists of recommendations, that can be overwhelming for already overstretched people in-country, to propose activities that build on and strengthen existing programmes and interventions in a structured way (see Fig. 2). This framework will be tested during responses to future TA requests.

### What worked well and what did not, and what are the implications of this for future action?

There have been a number of positive factors that facilitated and strengthened the technical support that has been provided during the first three years of the TA Mechanism, that have helped to ensure that it was timely, effective, efficient and contributed to strengthening capacity, as intended.

In particular, the way that different stakeholders mentioned below have worked together through the collaborative approaches that had been developed, both in the form of multi-person/multi-organizational teams, and also through efforts to build on existing in-country collaborations: ministries of health (in defining the TA requests), Partner Organizations (in working together to provide the requested TA), WHO regional and country offices (in maintaining ongoing communication with ministries of health) and the TA Mechanism Secretariat (in its facilitative and administrative roles) have all been important. The processes and principles included in the SOP similarly played an important role in shaping the day-to-day activities of the Mechanism, notably the development of an integrated TA plan and activities, that included ministries of health; regular meetings and communication, flexibility in terms of timing and approaches to TA, the commitment to involving young people and developing capacity, and the phased/long-term involvement with countries.

However, there have also been a number of challenges, and consequently the TA Mechanism has sometimes not worked exactly as originally planned, or hoped. These included the need for everyone to be clear about the purpose and functioning of the TA Mechanism (e.g., two requests for TA were essentially requests for funding for already-identified national consultants), and about the different roles and responsibilities for providing the TA, if these have not been well defined in the initial integrated TA plans and budgets. Some of the processes were considered to be very time consuming and needed to be further refined, for example the reviewing and commenting on outputs and contracting procedures. And in some cases the expectations for specific deliverables were unrealistic in terms of the time and resources available.

There were also concerns that sometimes insufficient attention was paid to involving national partners/consultants (although there have been encouraging experiences of this in several countries, for example Afghanistan, Malawi and Uganda), to involving young people in a meaningful way and to maintaining the engagement of the ministries of health that requested the TA - due to staff turnover, busy schedules, competing demands for their attention, and/or lack of pre-existing relationships between some TA providers and ministry of health counterparts. This may also have contributed to the finding that the TA Mechanism responses to date have paid too little attention to capacity development, despite the intention to do this.

There have also been several issues related to planning and implementation. Concerning planning, preparatory timelines were often unrealistic and not maintained. There were a number of reasons for this. For example, in several cases it took the TA providers time to fully understand the unspoken dynamics underpinning a request, the key stakeholders and other agencies who might be influencing the TA and the desired outcomes, and the final decision-makers. In addition, the development of tools and methods for data collection, analysis and prioritization took too long; there was sometimes a lack of clarity, or even disagreement about the focus of the TA (e.g. contraceptive uptake, ASRHR or adolescent health more generally); responsibilities and means for quality control were sometimes not adequately specified, including the fact that time for the TA Mechanism Secretariat to review tools and deliverables was not initially built into the timelines of the early TA responses; and the budget guidance was sometimes unclear, and the limited funding ceiling at times made things more complicated and caused some delays.

Concerning implementation, the Partner Organizations felt that there were sometimes too many meetings and processes that were too complex for the limited funding (e.g., to develop expressions of interest, and initial plans/budgets); and in general, not much attention was paid to potential risks and risk-mitigation. It was also found to be challenging to achieve sufficient cross-fertilization when multiple methodologies and partners are involved with the TA, and to define who has the final say when there are differences of opinion/perspectives. Likewise, it was sometimes difficult for both TA providers and people in the requesting countries to complete tasks in a timely way because of competing demands, compounded by individual and organizational changes, including those that took place within ministries of health.

Developing activities to strengthen the engagement of ministries of health has been one of the key changes that have taken place during the three years. This is reflected in the addition of two Annexes to the second version of the SOP, one that provides a structure for regular reports to the MOH and the other that clarifies what the TA Mechanism would (e.g. organizing inception and validation meetings) and would not (e.g. salaries) be willing to include for ministries of health in the overall integrated TA plans and budgets.

Based on the presentations and the subsequent discussions of the Review meeting that took place in June 2021, six issues were identified and discussed in detail. Table 3 provides details about activities that had already been implemented by the TA Mechanism in response to these issues, and outlines selected examples of further responses to these challenges that were proposed during the meeting.

**Table 3:** Key challenges and solutions [7]

**Challenge**	**Problem statements**	**Actions already implemented**	**Examples of Additional actions proposed**
**Who’s in the driver’s seat?**	While the Mechanism has worked hard to ensure that the TA requests are country-owned and driven, MOH leadership and continued engagement has been a challenge in some countries (e.g. with staff changes and competing priorities)	• Be explicit from the start that the TA is for the MOH and that the MOH is in the driver’s seat • Create space for the MOH to decide on the TA team, as long as it includes a Partner Organization • Identify clear roles and responsibilities for the MOH and provide contracts/budgets to support activities for which the MOH is responsible (e.g. the roles that JKP is playing in the Kenya TA) • Specify necessary data to support the TA and request assistance from MOH to obtain it (e.g. county-specific data) • Provide periodic updates to MOH about the TA (e.g. findings, requests for support, etc.) • Request feedback from MOH on specific activities/outputs	• Seek permission from MOH staff to contact them directly, if needed • Identify the “movers-and-shakers” in the country who can provide alternative channels of communication and insider-perspectives • Expand/nurture the role of the RO and WCO with the legitimacy/political clout to nudge progress • Establish a TA steering/coordination committee with relevant stakeholders in-country • Agree on focal points/decision-makers and their preferred ways of working (e.g. phone calls not emails) • Agree on timelines and regular check-in points, including realistic pause/restart (or pull-the-plug) timelines • MOH to be encouraged to propose potential partners, capacity development needs
**Too many cooks in the kitchen**	While collaboration between partner organizations was consistently noted as something positive and useful, it introduces challenges for leadership, coordination, and efficiency of the TA	• Spend time early in the process to develop clear roles and responsibilities, expectations, and timelines • Support creation of a “responsibilities matrix” (e.g. the planning process in Afghanistan, Malawi and Kenya) • Encourage regular (e.g. weekly) meetings to review progress/timelines/outputs (e.g. Malawi and Kenya)	• Find a balance between having equitable partners in the TA response and having one partner lead so that there is a “decision-maker” • The Secretariat to lean in to support collaboration for the TA but not to take on the routine facilitation role for ongoing activities • TORs to be drafted by TA Mechanism and agreed upon by TA team, with a clear responsibilities-matrix for all involved (i.e. Partner Organizations, MOH, youth organizations and other national stakeholders) • Find an effective balance between group consensus and individual productivity • Make sure that the different components of the TA are coordinated/integrated • Allow partnerships at the call for applications/expression of interest stage i.e. applicants can seek partnerships among the Partner Organizations • Explore systems for quality control of non-TA Mechanism partners/consultants
**Everything takes sooooo long**	While it is helpful to spend time up front to co-create and build consensus among stakeholders about the objectives and workplans, the preparatory work is sometimes too long and complicated for the relatively small awards	• Wait to request detailed proposals from Partner Organizations for a TA response until the TA team is formalized (e.g. Liberia) • Support creation of a “responsibilities matrix” (e.g. Afghanistan, Malawi, Kenya) • Have started the process for WHO Requests for Proposals (RFPs) which will allow for pre-qualification and higher budget ceiling (submissions will be requested from all Partner Organizations) • Developing a more flexible funding mechanisms, to counteract the budget limitations and allow more comprehensive thinking/strategy (in process)	• Use standard templates for workplans/budgets, while avoiding being too prescriptive • Share expected (and realistic!) timelines for project preparation and contracting – and stick to them! • Include risk assessment/mitigation planning in order to be aware of likely bottle-necks/delays: include ways to adapt to crises and competing responsibilities/timelines • Review TA Mechanism materials/SOP: are there opportunities to make the processes more flexible/streamlined • Be clear about the levels of detail that are required during initial assessments • identify "carrots and sticks" in relation to timelines • Consider having examples of methods for carrying out situation assessments, landscape analyses, programme reviews, etc. that can be adapted • Find an effective balance between group consensus and individual productivity!
**Acting Beyond tokenism**	While individual TA responses have incorporated elements of meaningful youth engagement, the Mechanism could and should do more	• Encourage Partner Organizations to identify opportunities for meaningful youth engagement (MYE) in the individual TA responses (e.g. consultation with young people as part of the prioritization exercise in Afghanistan, the involvement of youth committees in Kenya and Senegal) • Engage IYAFP as a Partner Organization and issue formal contract for their support to review/vet new TA requests and propose options for MYE in the responses	• Identify young people as consultants who could be engaged throughout the TA as part of the broader team - leveraging local or regional YP networks or committees already engaged with Partner Organizations • Use the TA to support MOHs to strengthen their partnerships with young people (capacity development) and build accountability mechanisms/frameworks into MOH activities • Bring young people into decisions about the development of their TA needs - the Partner Organizations who respond to TA requests also need to indicate clearly how young people are/will be involved in the delivery of the TA • Partner Organizations to include young people working in their organizations (e.g. affiliated youth champions/advocates, etc.) at the application and TA design stage, and engage the youth representative in national TWGs (where these exist) • Share preliminary findings of the TA with youth councils, youth-led organizations to see if we are missing any points that they believe are important
**Teaching people to fish**	While capacity development is an explicit objective of the TA Mechanism and has occurred to some extent, the types of capacity development and the outcomes of capacity development that are feasible and appropriate through TA provided over different periods of time should be further interrogated and systematically integrated in TA responses	• Encourage engagement from individuals at different levels of partner organizations: global, regional and national (e.g. Sierra Leone, Kenya, Senegal) • Engage local organizations/consultants as part of the TA team (e.g. Malawi) • Offer blended-learning courses on AYSRHR to country stakeholders (e.g. the Afghanistan MOPH participating in the Geneva Foundation for Training and Research MENA course)	• Encourage engagement from individuals at different levels of Partner Organizations: global, regional and national (e.g. Sierra Leone, Kenya, Senegal) • Engage local organizations/consultants as part of the TA team (e.g. Malawi) • Consider carrying out a needs assessment for capacity development at different steps of the TA during the initial planning phase (what is the capacity, what needs to be done by whom?) • Identify the key capacity gaps in order to target mentorship and sensitization sessions. • Capacity development/building needs to be intentional (a capacity transfer plan) - it should be a deliverable and it needs resources if it is to be done effectively
**The point of the spear**:	While the TA Mechanism tries to use adolescent contraception as the entry point to addressing AYSRHR more broadly, countries often want to do more and there have been challenges in defining the problem and/or the scope of the TA	• Support the MOH to clarify and confirm the problem(s) and objective(s) • Onboarding/introductory calls with various stakeholders at the start to reach consensus • Include key issues that go beyond but are related to ASRHR (e.g. nutrition in Afghanistan, HIV in Malawi)	• Identify opportunities to link with other on-going processes in countries • Support the MOH to better formulate the problem(s) and objective(s) and identify ways to include other adolescent health problems (e.g. mental health) • Onboarding call with all relevant parties (including the WHO country and regional team) to reach consensus • Where possible, include and/or link to other related areas (e.g. GBV) – but not too many of them! • Be clear about links between TA and other in-country processes for AYSRHR and adolescent health more generally

As a result of the experiences and lessons learned from the first two years of the TA Mechanism, the SOP was reviewed and a new version has been drafted

## Discussion

### What were our principal findings in relation to the questions we set out to answer?

Firstly, building on the limited available documentation on lessons learned in the provision of TA to strengthen health policies and programmes, and in consultation with key informants representing providers of TA, users of TA, and funding agencies that support the provision of TA, we designed a TA Coordination Mechanism that aimed to respond to the priorities of governments in LMICs – a mechanism with explicitly stated principles and a detailed *modus operandi* to try to avoid the limitations identified in current and previous TA provision efforts. Secondly, the Mechanism has demonstrated its feasibility, acceptability, and utility in filling some of the existing gaps in TA provision. Thirdly, the core of the TA Mechanism’s approach, i.e., working with a group of partner organizations with expertise in ASRH to collaboratively provide TA in line with an agreed upon set of principles and practices worked well. However, some of the processes that were put in place did not work as well as expected, and this has been outlined in the findings section. It has also been noted that efforts have been made on an ongoing basis to improve these processes and make them more fit for purpose.

The following factors were helpful: a funding agency willing to take a risk with this innovative approach, placing the TA Mechanism in WHO for both technical expertise and credibility, being realistic and forthright about what the TA Mechanism could achieve, open and ongoing communication, being flexible and finding ways to use challenging situations as opportunities. The slow pace of bureaucratic processes was clearly an internal hindering factor, as was the fact that traditional methods of TA provision are deeply ingrained and often set within unequal power relationships.

### How do the findings of our review of the TA Mechanism compare with those of other studies/evaluations/reviews of technical assistance?

First, there have been some attempts to evaluate the effectiveness of TA and to identify good practice and lessons learned in relation to different approaches to its provision. These include evaluations of TA, broadly defined and involving a range of projects and sectors, [8–10] assessments carried out by a range of partners [11–14] using different approaches [15] and focusing on different specific issues (for example, capacity building for staff of the ministry of health, NGOs and others, [16] programme evaluation [17] and the role of “sectoral advisers” [18]). Secondly, although specific mention of evaluating TA is sometimes missing from major TA providers, [19] there is a growing literature on the challenges of evaluating TA, [20] including the evaluation of TA within the context of broader organizational programme support, [21] and the development of concepts and guiding principles for the provision of TA [22, 23]. Thirdly, there have been attempts to assess and synthesize good practice in order to strengthen access to and use of TA [24, 25] and to identify lessons learned: for example, one study indicated that pursuing true country ownership for effective programmes requires long-term approaches involving persistence, patience, keen understanding of counterparts’ perspectives, deference, building trust, a focus on priorities, technical competence, and sustained optimism [26]. One of the more detailed and systematic reviews of TA provision explored, among other things, the costs of different approaches to providing TA; and stressed the importance of moving beyond “one-off interventions”, moving from international staff to well-trained national staff (to decrease the major costs of personnel and travel for TA, but also for increasing the contextual relevance of the TA and to enhance national ownership); using internet-based approaches more effectively and focusing TA on evidence-based priorities (rather than trying to respond to every “pull request”) [27]. The impression we take away is that overall, efforts to study/review/evaluate TA are not commensurate with the resources that are invested in providing or paying for TA.

### What are the implications of the findings of our review and our learning from the work of others for action and research?

Understanding and navigating system constraints: The literature review and key informant interviews that informed the development of the TA Mechanism identified a number of challenges in existing TA practice. Although the TA Mechanism aimed to address these challenges, a number of them persisted. These included the declining engagement of ministries of health over time; limited capacity development and engagement of young people; mismatches in expectations between the ministry of health and the TA Mechanism; and limited follow-up actions on the uptake of TA Mechanism deliverables (e.g., in terms of the operationalization of the guidelines that were developed in Sierra Leone). The TA Mechanism Secretariat and partners were aware of these challenges, so the fact that these problems continued in some of the countries is not due to a knowledge gap. Rather, they are likely to be a reflection of the constraints and complexities of the national and global health systems and structures that the TA Mechanism is operating in, and the influence of past ways of doing things. Going forward, it will be important to explore what systems factors hamper the impact of TA, even in the face of better processes for requesting and providing TA. Systematically identifying these factors could inform adaptations to both the impact model and TA processes in general.

Sharing and adapting tools and lessons learned: Although a protocol was developed in the Mechanism’s SOP for assessing tools that might be used in providing TA, one of the things that was not done during the first three years of the TA Mechanism’s work was to develop a repository of tools that Partner Organizations had found to be useful for rapid programme reviews, landscape analyses, key informant interviews and other components of situation assessments. Furthermore, a number of new tools have been developed by Partner Organizations and the TA Mechanism Secretariat that could also be made more widely available, for use and adaptation by others (e.g., the Opportunities Framework). In addition to programme support tools, it will be important to share the lessons learned about providing TA that have been gained from the TA Mechanism (this paper is a start), to develop more in-depth analyses of experiences in specific countries (initial discussions have taken place to do this for Afghanistan and Kenya), and to link with other initiatives that are exploring new approaches to providing TA, to share ideas and experiences - this has also been started.

Partnerships: The development and nurturing of partnerships has been central to the development of the TA Mechanism, both internally (e.g., the collaborations between the Partner Organizations) and externally (e.g. between the TA Mechanism and partners such as FP2030 and the funder, BMGF). However, additional strategic partnerships will be important to nurture during the coming years, including with UN organizations that have key roles to play in relation to AYSRHR in countries. In addition, partnerships with young people need more attention, to take advantage of the many opportunities that the TA Mechanism provides to strengthen their engagement: from including them in the development of TA requests and proposals to ensuring their meaningful involvement in the provision of TA, for example facilitating key informant interviews and participating in Inception and Validation meetings.

The need for different approaches for quality control: One of the things that the TA Mechanism sought to do from the start was to give adequate attention to quality assurance of the processes and outputs of the TA that was provided. However, the time and effort required to do this was significantly underestimated by the TA Mechanism Secretariat. As the number of countries increase, the regional and headquarters staff of Partner Organizations will need to play an increasingly important role in maintaining the quality of the TA provided. In addition, preliminary discussions have been held on developing a small group of consultants who are experts in the field and could be on hand to assist with reviewing deliverables, documents and reports, and providing feedback.

Technical capacity in countries: As both the evidence base for action and the technical capacity in countries requesting TA have become stronger, it will be increasingly important to ensure that TA responses make a contribution to mentoring and capacity strengthening, and that they move from focusing on the “what needs to be done?” type questions to dealing more explicitly with “how to do what needs to be done, in different contexts and for different groups?” In many settings it needs to be recognized that sometimes “technical facilitation” is perhaps more needed than technical assistance *per se*. However, it will also be important to be clear about what type of capacity development might be feasible and desired by ministries of health and national counterparts in the context of sometimes short-term, deliverable-driven TA.

From process to impact: Moving forward, it will be important for the TA Mechanism to work towards answering a number of questions that are currently not possible to answer. This includes questions such as: “what was the effect of the TA Mechanism – did the outputs of the TA contribute to the desired outcomes and impact?” and “were the outputs of the TA Mechanism of use to the country – was the TA Mechanism’s work used, and was it useful?” To accomplish this, more effort will need to be paid to realizing the TA Mechanism’s initial intentions, as outlined in the SOP, to monitor the TA that has been provided in terms of a range of inputs, processes and outputs, including milestones and quality; and to develop systems that would systematically evaluate the outcomes and the costs of the TA that is provided, both quantitatively and qualitatively. This will be a significant challenge for the coming years.

TA as a contribution to broader public health agendas: This paper provides an overview of the lessons learned from WHO’s AYSRHR TA Coordination Mechanism, which was developed in response to the technical assistance gaps that face some ministries of health in terms of moving from want-to-act to action, by providing country-driven TA that is timely, efficient, effective and contributes to strengthening capacity. It is to be hoped that in addition to helping stimulate improvements in the functioning of the TA Mechanism, this synthesis of the lessons learned from the TA Mechanism during its first three years of functioning will contribute both to wider discussions about approaches to the provision of technical support to LMICs, including the Impact Model and the Opportunities Framework, at a time when capacity in these countries is rapidly strengthening; and also to discussions around efforts to shift to stronger country-driven global health agendas.

### What are the strengths and weaknesses of our study?

The strengths of this study are as follows: firstly, it includes the perspectives of two key stakeholders – those who requested and received the TA, and those who responded to these requests; secondly, it has been developed with the inputs of the Partner Organizations and other key collaborators; and thirdly, it seeks to rapidly share lessons learned, both for the TA Mechanism and for the wider public health community – to generate discussion and strengthen the evidence-base on TA provision. Its limitations are as follows: firstly, it is still early in the process of implementing the TA Mechanism and as such it is only possible at this stage to review process and outputs, not yet the outcomes and impact of the TA provided; secondly, it has mostly been carried out by the TA Mechanism Secretariat, rather than by someone external; and thirdly, it is a review of where we are and what needs to be changed, rather than a structured evaluation based on quantitative and qualitative data (which is planned for at a later stage), which could certainly include some biases.

## Conclusions

Despite their growing capacity in AYSRH, many countries need TA on different aspects of their policies and programmes. Current mechanisms to provide TA have a number of limitations. The AYSRH TA Mechanism was set up with funding from the Bill and Melinda Gates Foundation in WHO to address this challenge. It set out to provide countries with the technical assistance they need – with Ministries of Health in the drivers’ seat, drawing upon expertise that was available in and around countries seeking support, and using approaches that built capacity in the global South. In its first three years of operation, the AYSRH TA Mechanism has shown that is feasible, acceptable to different stakeholders, and provides examples of different ways of providing TA that attempt to avoid some of the limitations of traditional approaches. These findings, from reports and meetings with stakeholders, need to be validated with an independent evaluation. The global public health and development community needs to invest more time and effort in learning through doing, to make TA as good and effective as it should be, relative to the large investments in TA that are made by most organizations.

## Data Availability

All referenced unpublished reports available from the authors on request.

## Abbreviations

ADH: Adolescent Health
AKU: Agha Kahn University
ASRH: Adolescent Sexual and Reproductive Health
BMGF: Bill and Melinda Gates Foundation
CSO: Civil society organization
EGPAF: Elizabeth Glazer Pediatric AIDS Foundation
Equipop: Equilibres et Population
IPPF: International Planned Parenthood Federation
IYAFP: International Youth Alliance for Family Planning
JKP: Jumuiya ya Kaunti za Pwani (in Swahili) meaning Commonwealth in English
LGA: Local government area
LMIC: Low- and middle-income country
MAMTA: MAMTA Health Institute for Mother and Child
M&E: Monitoring and evaluation
MOH: Ministry of Health
MOHP: Ministry of Health and Population
MOPH: Ministry of Public Health
NGO: Non-governmental organizations
PSI: Population Services International
RMNCAH: Reproductive, Maternal, Newborn, Child and Adolescent Health
SDGs: Sustainable Development Goals
SOP: Standard operating procedures
SRH: Sexual and reproductive health
TA Mechanism: WHO AYSRHR Technical Assistance Coordination Mechanism
TOR: Terms of reference
TWG: Technical Working Group
UHC: Universal health coverage
UNAIDS: United Nations Joint and Co-sponsored Programme on HIV/AIDS
UNFPA: United Nations Fund for Population
UNICEF: United Nations Children Fund
WCO: WHO Country Office
WHO SRHR: World Health Organization’s Department of Sexual and Reproductive Health and Research
